# The use of optical coherence tomography (OCT) and OCT angiography in borderline personality disorder compared to health control subjects

**DOI:** 10.1111/cns.14699

**Published:** 2024-03-26

**Authors:** Bei Xu, Fangling Li, Zhejia Zhang, Qian Xiao

**Affiliations:** ^1^ Eye Center of Xiangya Hospital Central South University Changsha China; ^2^ Hunan Key Laboratory of Ophthalmology Central South University Changsha China; ^3^ National Key Clinical Ophthalmology Specialist, Xiangya Hospital Central South University Changsha China; ^4^ Hunan Provincial Branch of the National Clinical Medical Research Center for Eye Diseases, Xiangya Hospital Central South University Changsha China; ^5^ National Clinical Research Center for Geriatric Disorders, Xiangya Hospital Central South University Changsha China; ^6^ Department of General Surgery, Xiangya Hospital Central South University Changsha Hunan China; ^7^ Mental Health Center of Xiangya Hospital Central South University Changsha Hunan China

**Keywords:** borderline personality disorder, OCT angiography, optical coherence tomography angiography, perfusion density

## Abstract

**Background:**

Optical coherence tomography (OCT) or OCT angiography (OCTA) has been investigated in few research studies of psychiatric disorders. No research has been done using OCT or OCTA in patients with borderline personality disorder (BPD).

**Methods:**

OCTA measured foveal avascular zone (FAZ), macular vessel density (MVD), and peripapillary vessel density (PVD). OCT measured the peripapillary retinal fiber layer (RNFL) and central retinal thickness (CRT). The study utilized the Ottawa Self‐Injury Inventory, Hamilton Anxiety Rating Scale (HAMA), and Global Assessment of Functioning (GAF) to assess the symptom characteristics of individuals with BPD.

**Results:**

Fifty‐nine eyes of BPD patients and 58 eyes of normal subjects were analyzed, MVD of the superficial retinal capillary plexus declined noticeably in most subfields (*p* < 0.05). Significant differences were observed in the whole inner ring and outer ring index between BPD and HC groups (*p* < 0.05). The patients with BPD exhibited lower RNFL and CRT, the difference was significant (*p* < 0.05). CRT indicated a significant negative correlation with the Ottawa Self‐Injury Inventory (*p* < 0.05). In addition, we observed that there was a negative correlation identified between the MVD of the inner ring and HAMA (*p* < 0.05). Meanwhile, the MVD of the outer ring was positively correlated with GAF (*p* < 0.05). The areas under the receiver operating characteristic curves (AUROCs) for distinguishing BPD and HC eyes in OCTA were the highest for fovea MVD (0.679), followed by outer ring MVD (0.669), inner ring MVD (0.641), FAZ (0.579). In OCT, CRT was highest for BPD (0.711), followed by RNFL (0.625).

**Conclusion:**

The OCT and OCTA can non‐invasively detect microvascular and morphology changes of the retina in BPD patients compared to healthy control subjects.

## Graphical abstract

Optical Coherence Tomography recognition of Borderline Personality Disorder and Healthy Controls eyes, the area under the receiver operating characteristic curve was highest for fovea, followed by outer ring, inner ring, foveal avascular zone, central retinal thickness and peripapillary retinal fiber layer.

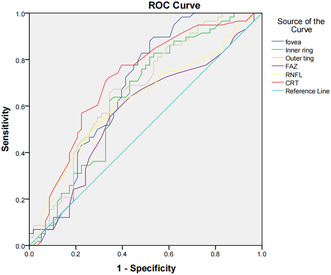

## 1 INTRODUCTION

Borderline Personality Disorder (BPD) is a complex psychological disorder characterized by emotional instability, unclear self‐image, unstable interpersonal relationships, impulsivity, and non‐suicidal self‐injury behaviors (NSSI).^1^ It is estimated that the prevalence of BPD in the general population is around 5%, while it can be as high as 10% in psychiatric clinics.^1^

Finding neural mechanisms for BPD is an important research direction because it can help us better understand the pathophysiological mechanisms of BPD. Therefore, finding a simple, effective, and rapid method to identify neural abnormalities is crucial. Some studies have found that patients with BPD may have chronic inflammatory responses and neuronal damage, which may be related to abnormalities in the hypothalamic–pituitary–adrenal axis function.^2^ Some researchers suggest that young patients with comorbidities of BPD and MDD have more obvious endocrine and immune abnormalities than young patients with MDD alone.^3^ Recently, thinning of the nerve fiber layer and retinal vein dilation have been reported in patients with psychiatric disorders.^4^, ^5^ Both studies contribute to the growing evidence that retinal vascular measures, especially retinal venular diameter, could reflect brain structural abnormalities in schizophrenia.^4^, ^5^ They highlight the potential of using neuroretinal biomarkers as non‐invasive indicators of neurodevelopmental abnormalities associated with schizophrenia.

Optical Coherence Tomography (OCT) is a non‐invasive imaging technique that uses light waves to measure the microstructures of the retina, widely used in diagnosing ophthalmic diseases. Optical coherence tomography angiography (OCTA) uses OCT to generate images by interfering with reflected light.^6^ It can measure tissue density and structure in each layer of the retina, as well as display various sizes of vascular networks, including capillaries.^6^ Compared with traditional retinal imaging techniques, such as fluorescein angiography, OCTA does not require contrast agents to be injected, and it does not cause any adverse reactions in patients. As a non‐invasive imaging technology, OCTA has the advantage of evaluating vascular abnormalities in multiple diseases and therefore has broad prospects for application in the medical field.^7^ Interestingly, OCTA has been used for researching psychiatric disorders (schizophrenia, bipolar disorder, and major depressive disorder) in recent years.^8^, ^9^, ^10^ All three psychiatric disorders show retinal changes detectable through OCT and OCTA. This suggests a potential common pathway in these psychiatric disorders where retinal microvascular changes are involved. In patients with major depressive disorder, OCTA has shown reduced retinal vessel density and thinning of the subfoveal choroid.^9^, ^10^ Patients with schizophrenia and bipolar disorder have demonstrated microvascular dysfunction in the retina, especially in its central part.^8^ Although, there is a lack of OCTA or OCT research on BPD, and the relationship between BPD and retinal microvascular density and morphology is still unclear.

This study is the first to use OCTA and OCT to explore BPD, and we recruited 48 BPD patients and 35 healthy controls (HCs) to quantitatively evaluate changes in retinal vascular density and structure in patients with BPD, exploring changes in retinal indicators and the OCT combined with OCTA parameters for BPD. In addition, we will also investigate the relationship between these retinal indicators and symptoms of BPD, including NSSI, anxiety, and functional impairment.

## 2 METHODS

### 2.1 Participants

The study abided by the Declaration of Helsinki, which was performed by complying with the approved guidelines and regulations. approved this study, and all participants provided written informed consent. We enrolled 48 adults with BPD and 35 age‐ and gender‐matched HCs. Patients with BPD were consecutively recruited from the Psychiatric Clinics at the Mental Health Center of Xiangya Hospital from December 2021 to December 2022. HCs were recruited from advertisements during the same study period. To be included in the patient group, individuals had to meet the following criteria: (1) be between 18 and 45 years of age, (2) meet the diagnostic criteria for BPD outlined in the DSM‐IV,^11^ and (3) have had stable symptoms for more than 2 years, with no better explanation by DSM‐IV Axis I psychiatric or neurodevelopmental disorders. The HCs had to meet the following criteria: be between 18 and 45 years of age, and have no history of psychiatric disorders or psychotropic medication. Exclusion criteria for all participants were as follows: history of schizophrenia spectrum disorder, bipolar spectrum disorder, post‐traumatic stress disorder, neurodevelopmental disorders, major depressive disorder, alcohol or drug dependence, neurological disorders, or an intelligence quotient (IQ) of ≤80. All participants have not taken any psychotropic, vascular, or ophthalmic medications for at least 4 months. Besides, all participants were asked to avoid alcohol or psychotropic substances for 24 h before the OCTA. The ophthalmic exclusion criteria were presented as the patients with (1) glaucoma, age‐related macular degeneration, macular hole, epiretinal membrane, refractive error [> ±3 diopters (D) sphere and ±2D cylinder], macular drusen, as well as pathological myopia, (2) diabetes and uncontrolled hypertension, (3) concomitant neurological disorders, (4) severe cardiac, pulmonary, hepatic, renal diseases, or any types of tumors, and (5) insufficient clinical data.

### 2.2 Structured interview and symptom assessment

To confirm the diagnosis of BPD, a structured interview was conducted using the BPD section of the Structured Clinical Interview for DSM‐IV Personality Disorders (SCID‐II).^11^ Confirmation of the diagnosis required five or more of the nine diagnostic items. To assess for Axis I mental disorders, the Structured Clinical Interview for DSM‐IV Axis I Disorders (SCID‐I) was used.^12^ The same structured interviews were administered to the HCs to rule out personality disorders and Axis I mental disorders. The final diagnosis was made by a clinical team consisting of two senior psychiatrists (Q.X and F.J) with 10 and 8 years of subspecialty experience in psychiatry, respectively. A comprehensive clinical evaluation was performed.

A skilled psychiatrist (FJ) with 8 years of experience in psychiatric evaluation performed the clinical assessment and interviews. This study utilized the Ottawa Self‐Injury Inventory to assess the characteristics of NSSI behavior based on the scales of function and addiction.^13^ Subscale 4A of the Ottawa Self‐Injury Inventory corresponds to the social impact function of NSSI, subscale 4B of the Ottawa Self‐Injury Inventory represents the external emotion regulation function of NSSI, and subscale 4C of the Ottawa Self‐Injury Inventory represents the internal emotion regulation function of NSSI. Subscale 5 of the Ottawa Self‐Injury Inventory measures the addictive features of NSSI. In this study, the Hamilton Anxiety Rating Scale (HAMA)^14^ was employed to assess anxiety levels in BPD patients, while the Global Assessment of Functioning (GAF)^15^ was used to evaluate the overall level of functioning in patients. All interviews and scale evaluations were conducted on the same day as the OCTA examination.

### 2.3 Ophthalmic examination

All participants received a complete ophthalmic examination including BCVA, and intraocular pressure (IOP) measurement with Goldmann applanation tonometry. All participants underwent OCTA and OCT imaging based on the Cirrus 5000 HD‐OCT (Carl Zeiss Meditec, Inc., Dublin, CA, USA). The MVD and PVD in superficial retinal capillary plexus (SRCP) were analyzed throughout the examined area of 6 mm × 6 mm that was centered on the macula and peripapillary, and they consisted of vessels from the layer of the inner limiting membrane (ILM) to the inner plexiform layer. In addition, the vessel density was measured in nine subfields of the Early Treatment Diabetic Retinopathy Study grid (e.g., a central 1‐mm circle), respectively, representing the foveal area, and inner and outer rings of diameters 3 and 6 mm. The inner and outer rings fell to four quadrants, i.e., superior, nasal, inferior, and temporal. The foveal avascular zone (FAZ) area was automatically segmented and then quantified with the OCTA software. Central retinal thickness (CRT) was represented as the average thickness of the 1 mm ring in the center of the retina, and peripapillary RNFL was measured by an automatic analysis algorithm of OCT. To guarantee the integrity of the images, each OCT and OCTA image underwent a manual quality review by an experienced ophthalmologist (X.B). Eyes were scanned on a random basis, taking into consideration the time available. It is required that the pupil be adequately dilated at least 30 min before the examination. Each eye received two examinations, ensuring only high‐quality images were included. Images with suboptimal signal strength (signal strength index <7) or visible motion artifacts were discarded. The person assessing the images was not aware of the participants' clinical diagnoses. These details have been incorporated into the methods section.

### 2.4 Statistical analysis

Statistical analysis was performed using SPSS version 25.0. Gender differences were compared using the Pearson Chi‐square test. Continuous variables with a normal distribution were expressed as mean ± standard deviation and were analyzed using the Student's *t*‐test to compare differences between the BPD and HC groups. Continuous variables that did not follow a normal distribution were expressed as median (25% quartile, 75% quartile) and were analyzed using the Kruskal‐Wallis test. All data in this article conform to a normal distribution, and the Shapiro–Wilk test was employed to assess normality. Correlations between data were tested using Spearman's correlation. Statistical significance was defined as *p* < 0.05.

## 3 RESULTS

**FIGURE 1:**
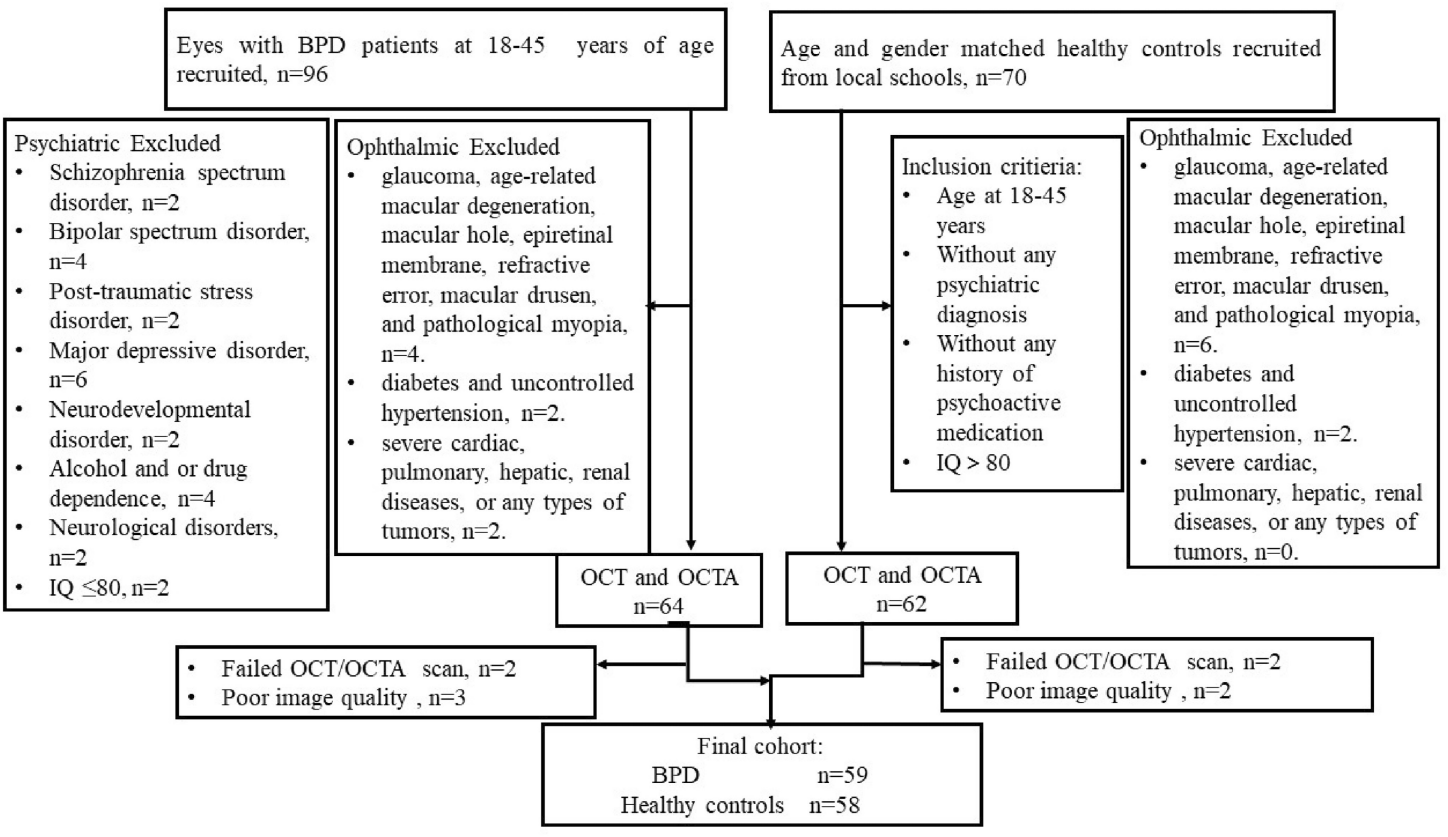
Flow chart illustrating the enrolment process. BPD, Borderline Personality Disorder; OCT, Optical Coherence Tomography; OCTA, Optical Coherence Tomography Angiography.

A total of 83 participants were recruited in this study, including 48 BPD patients (96 eyes) and 35 healthy controls (70 eyes). Some participants were excluded due to exclusion criteria for psychiatric disorders, ocular conditions, failure to complete OCT/OCTA scans, and poor image quality. Ultimately, analysis was conducted on 59 eyes of BPD patients and 58 eyes of normal controls. Poor image quality was attributed to the eye movement in most cases (Figure 1). No statistical difference was identified in age distribution (*p* = 0.949), sex (*p* = 0.650), education (*p* = 0.916), intraocular pressure (IOP) (*p* = 0.454), best‐corrected VA (BCVA) (*p* = 0.425) between BPD and HC group (Table 1). Furthermore, the patients with BPD exhibited lower RNFL (*p* = 0.021) and CRT (*p* = 0.001). The Ottawa Self‐Injury Inventory, HAMA, GAF scores, as well as the duration of illness and age of onset for the BPD patient group, are presented in Table 1. Table 1 lists the general demographics, ophthalmic features of all participants, and clinical parameters.

**TABLE 1:** Clinical characteristics of all included eyes.

	BPD (*n* = 59)	HC (*n* = 58)	*t*/χ^2^	*p*
Sex			0.205	0.650
Male	23	25		
Female	36	33		
Age (year)	20.4 ± 3.71	19.8 ± 3.54	0.067	0.949
Education	12.8 ± 4.06	13.1 ± 3.98	0.109	0.916
Illness duration (year)	4.5 ± 4.11	—	—	—
Onset age (year)	15.9 ± 3.16	—	—	—
Ottawa Self‐Injury Inventory‐raw score	25.63 ± 12.21			
Ottawa Self‐Injury Inventory‐4A	5.53 ± 5.02			
Ottawa Self‐Injury Inventory‐4B	11.13 ± 5.33			
Ottawa Self‐Injury Inventory‐4C	8.97 ± 5.27			
Ottawa Self‐Injury Inventory‐5	8.33 ± 7.48			
HAMA	20.10 ± 9.04			
GAF	55.77 ± 9.79			
BCVA (logMAR)	−0.09 ± 0.07	−0.10 ± 0.06	0.801	0.425
IOP (mmHg)	15.6 ± 2.08	15.3 ± 2.00	0.751	0.454
RNFL (μm)	103.3 ± 7.71	106.6 ± 7.3	−2.337	0.021
CRT (μm)	281.8 ± 13.82	289.7 ± 10.70	−3.457	0.001

Abbreviations: BCVA, best corrected visual acuity; CRT, Central retinal thickness; GAF, Global Assessment of Functioning; HAMA, Hamilton Anxiety Rating Scale; IOP, intraocular pressure; RNFL, retinal nerve fiber layer.

### 3.1 Macular vessel density parameters

Table 2 presents the macular vessel density (MVD) parameters in SRCP of all participants. The results demonstrated that compared with HC groups, MVD in the BPD group declined noticeably in all subfields (*p* < 0.05) except SO area (*p* = 0.066) (Table 2). Moreover, quadrantal analyses indicated the FAZ area decreasing in BPD patients in comparison with the HC group [0.29 ± 0.07 mm^2^ in healthy eyes vs. 0.27 ± 0.07 mm^2^ in patients], but the difference was not significant (*p* = 0.134) (Table 2). Moreover, specific to the whole inner ring and outer ring index, statistically significant differences were reported for the BPD and HC group (*p* < 0.05) (Table 2).

**TABLE 2:** Superficial retinal capillary plexus (SRCP) vessel densities in the BPD patients and healthy controls (HC) eyes.

	BPD	HC	*t*	*p*
Fovea	7.8 ± 3.02	9.5 ± 1.61	−3.633	<0.001
SI	17.2 ± 3.22	18.3 ± 1.17	−2.424	0.017
II	17.2 ± 2.44	18.4 ± 1.14	−3.268	0.002
NI	17.6 ± 2.10	18.5 ± 1.10	−2.903	0.005
TI	17.1 ± 2.27	18.4 ± 1.05	−3.345	0.001
SO	18.5 ± 1.12	18.9 ± 0.72	−1.858	0.066
IO	17.9 ± 1.57	18.8 ± 0.80	−4.011	<0.001
NO	19.6 ± 1.04	20.0 ± 0.74	−2.164	0.033
TO	16.5 ± 2.16	17.5 ± 1.15	−2.986	0.004
FAZ	0.27 ± 0.07	0.29 ± 0.07	−1.509	0.134
Inner ring	17.1 ± 3.17	18.4 ± 0.95	−3.042	0.003
Outer ring	18.1 ± 1.17	18.8 ± 0.61	−3.716	<0.001

Abbreviations: FAZ, Foveal avascular zone; II, Inferior inner; IO, Inferior outer; NI, Nasal inner; NO, Nasal outer; SI, Superior inner; SO, Superior outer; TI, Temporal inner; TO, Temporal outer.

### 3.2 Peripapillary vessel density parameters

The PVD parameters of all participants are presented in Table S1. As indicated from the result, no significant difference of PVD was found in all the subfields of the peripapillary scan between BPD and HC groups (Table S1). However, the MVD in all grids in patients with BPD were lower than those in HC.

### 3.3 Correlation between OCT and OCTA parameters and clinical parameters

**FIGURE 2:**
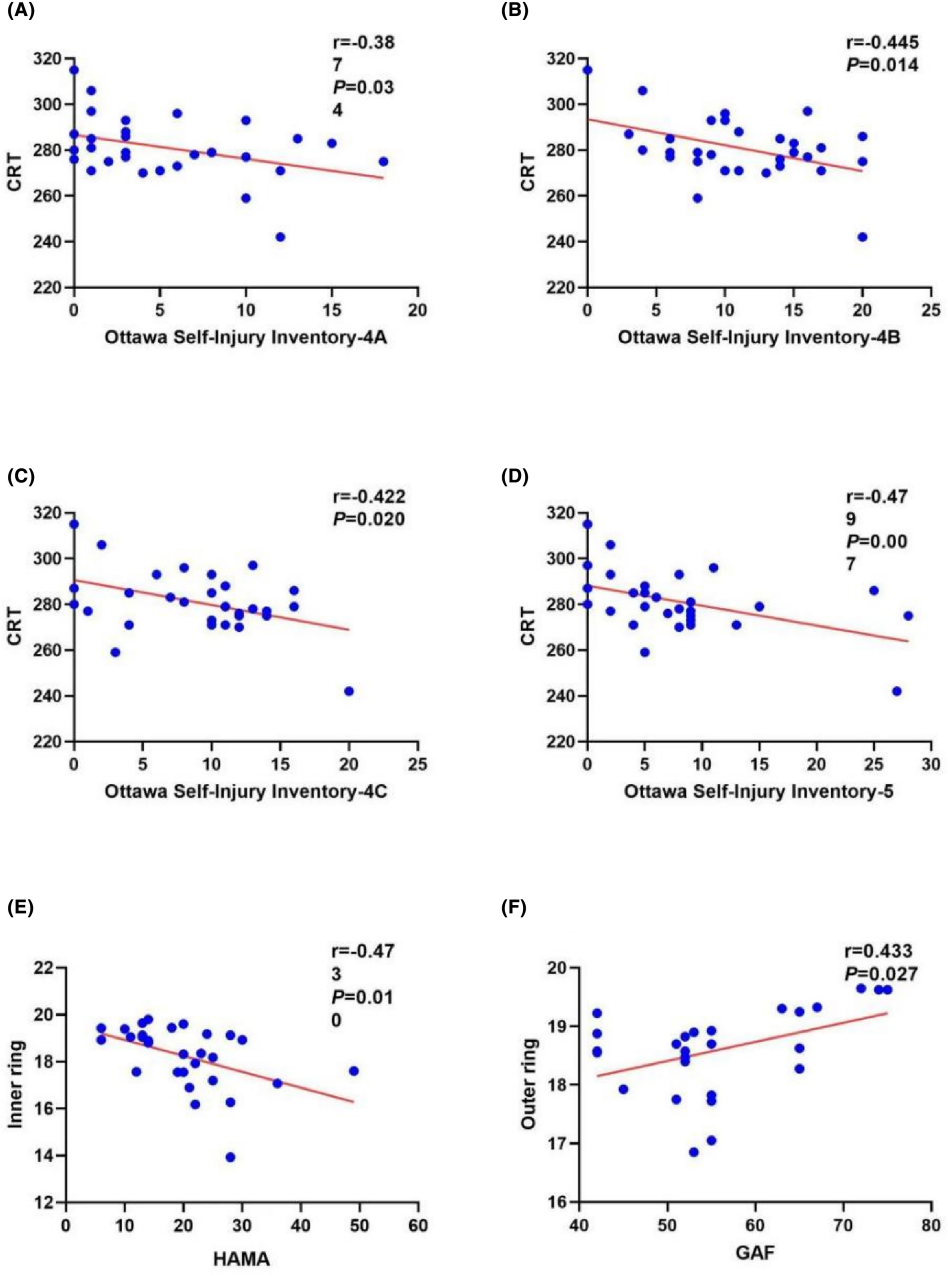
Correlation analysis between OCT and OCTA parameters and the scores for Ottawa Self‐Injury Inventory‐4A, Ottawa Self‐Injury Inventory‐4B, Ottawa Self‐Injury Inventory‐4C, Ottawa Self‐Injury Inventory‐5, HAMA, GAF in the patients with BPD. (A) Negative correlation between the CRT and the Ottawa Self‐Injury Inventory‐4A. (B) Negative correlation between the CRT and the Ottawa Self‐Injury Inventory‐4B. (C) Negative correlation between CRT and the Ottawa Self‐Injury Inventory‐4C. (D) Negative correlation between CRT and the Ottawa Self‐Injury Inventory‐5. (E) Negative correlation between the MVD of the inner ring and HAMA. (F) Positive correlation between the MVD of the outer ring and GAF. The threshold was set at a significance level of *p* < 0.05 (uncorrected). BPD, borderline personality disorder; OCT, Optical Coherence Tomography; OCTA, Optical Coherence Tomography Angiography; CRT, Central retinal thickness; MVD, Macular vessel density; HAMA, Hamilton Anxiety Rating Scale; GAF, Global Assessment of Functioning.

The correlation of MVD of patients with BPD with clinical parameters was analyzed, and Spearman's correlation coefficients and corresponding *p*‐value are presented in Figure 2. As indicated from the results, CRT indicated a significant negative correlation with Ottawa Self‐Injury Inventory‐4A (*r* = −0.38, *p* = 0.03), Ottawa Self‐Injury Inventory‐4B (*r* = −0.445, *p* = 0.014), Ottawa Self‐Injury Inventory‐4C (*r* = −0.422, *p* = 0.020), and Ottawa Self‐Injury Inventory‐5 (*r* = −0.47, *p* = 0.007). In addition, we observed that there was a negative correlation identified between the MVD of the inner ring and HAMA (*r* = −0.47, *p* = 0.010). Meanwhile, the MVD of the outer ring was positively correlated with GAF (*r* = 0.433, *p* = 0.027).

### 3.4 Distinguishing abilities of OCTA and OCT

**FIGURE 3:**
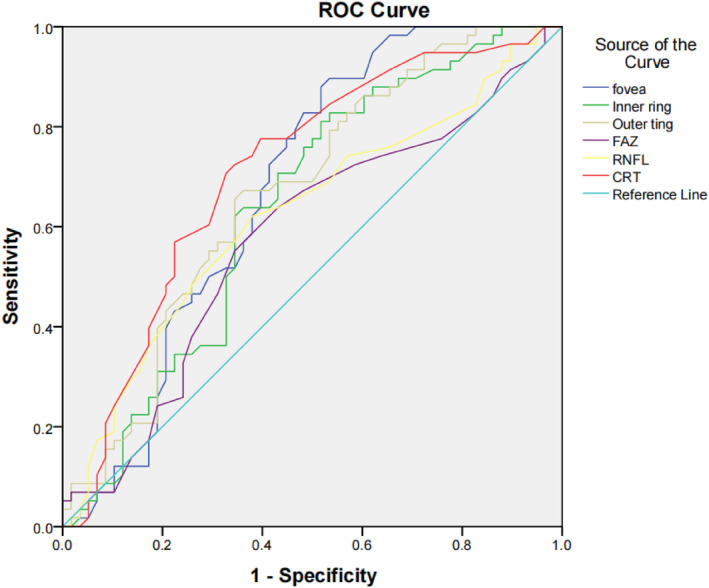
The areas under the receiver operating characteristic curves (AUROCs) for distinguishing BPD and HC eyes. RNFL, retinal nerve fiber layer; CRT, Central retinal thickness; FAZ, Foveal avascular zone.

The areas under the receiver operating characteristic curves (AUROCs) for distinguishing BPD and HC eyes in OCTA were the highest for fovea MVD (0.679), followed by outer ring MVD (0.669), inner ring MVD (0.641), and FAZ (0.579). In OCT, CRT was highest for BPD (0.711), followed by RNFL (0.625) (Figure 3).

## 4 DISCUSSION

To the best of our knowledge, this study is the first to provide OCTA and OCT analyses of the retina in patients with BPD. We found that BPD patients had significantly decreased MVD in the SRCP compared to the control group. Additionally, we observed significantly reduced foveal center retinal thickness (CRT) and retinal nerve fiber layer (RNFL) thickness in the BPD group compared to the control group. The area of the FAZ was decreased in BPD patients compared to the healthy group, but the difference was not significant.

OCTA was introduced in 2015, and since then, there has been an increasing amount of available research data. OCTA is a novel non‐invasive quantitative retinal imaging tool.^7^ OCTA‐ and OCT‐based parameters of the retina, such as retinal vessel density and thickness, are expected to become one of the most promising and competitive non‐invasive indicators in clinical practice. The retina is the only visualized neurovascular tissue in the body.^7^ The pathophysiology of many central nervous system disorders is related to alterations in the microvascular system. OCTA has been used to describe vascular abnormalities in several neurological diseases, such as multiple sclerosis, Alzheimer's disease, Leber's hereditary optic neuropathy, Parkinson's disease, Huntington's disease, amyotrophic lateral sclerosis, and migraine.^16^ However, these study results suggest that the OCTA method is still in its initial stages, with many contradictory findings. Besides, the number of reports associating OCTA examinations with psychiatric disorders is still limited. In comparison to previous studies, our research on BPD shows distinct findings. While major depressive disorder was associated with lower retinal vessel density, thinner retinal superficial macular layers, and poorer visual function,^9^ and schizophrenia and bipolar disorder were linked to significantly lower vessel density and macular thickness in the deep capillary complex,^8^ our study on BPD indicates different aspects of retinal vascular damage. Specifically, we found lower MVD in the SRCP of the macula, CRT, thinner RNFL thickness, and a decreased FAZ area.

Previous studies have also revealed retinal changes in various psychiatric disorders,^8^, ^9^ but the specificity of these findings for BPD remains unclear. Therefore, while our study is scientifically significant, its clinical application is yet to be determined. Future research should focus on exploring the specificity of OCT/OCTA abnormalities to determine if they are non‐specific across different psychiatric and neurological conditions.

This study identified a negative correlation between CRT and the functional aspects of NSSI (social impact function, external emotion regulation function, and internal emotion regulation function) as well as anxiety. This association suggests that lower CRT is associated with greater severity of NSSI. Additionally, the study found an association between CRT and the addictive nature of NSSI. Addictive behaviors are considered a means of emotional and self‐regulation, which is relevant to BPD patients who often engage in NSSI as a way to cope with emotions.^1^ Hence, the association between CRT and the addictive nature of NSSI may stem from their shared reflection of impaired emotion regulation strategies. Furthermore, there was a positive correlation between CRT and overall functional level in patients, indicating that thinner CRT is associated with poorer overall functioning. In fact, this study revealed a close association between CRT and these symptoms of BPD, suggesting that CRT can be used to predict the severity of BPD.

We speculate that changes in MVD may precede structural alterations, meanwhile, The vascular density in the macular region may undergo changes before the vascular density around the optic disc. According to the results no significant difference of PVD was found in all the subfields of the peripapillary between BPD and HC groups. The main reason for this phenomenon is likely the fact that the macular SRCP primarily originates from the retinal circulation, which aligns with the findings of this study. However, the radial peripapillary capillary receives perfusion from both the retinal circulation and the choroidal ciliary vessels.^17^ Previous studies have indicated that there is a concentration of thinner nerve fibers on the temporal side of the optic nerve head. This thinning of nerve fibers is associated with a shallower decline in the RNFL.^18^ This study is premature to conclude that these results can effectively guide early screening and efficacy assessment of BPD. Further studies are needed to more fully evaluate the clinical applications and limitations of these findings. However, the translation of theory into practice still faces many challenges.

Our study has significant limitations that impact the utility of OCT/OCTA as a diagnostic biomarker for BPD. The small sample size and high female proportion (61%) may introduce gender bias and limit the generalizability of our findings. Notably, the predominance of female BPD patients hinders the inclusion of a substantial male cohort. However, in the real world, BPD is more prevalent among women than men. The cross‐sectional nature of our study restricts our ability to observe longitudinal changes in retinal microvascular parameters. Variability in blood flow and vascular anatomy could also affect our results. Importantly, we did not account for variations in disease duration, and existing literature presents inconsistent findings regarding the relationship between RNFL thickness and psychiatric disorder duration.^19^, ^20^ Critically, our observations of OCTA abnormalities in BPD closely resemble those in depression, raising concerns about their specificity to BPD. This study lacks a comparative analysis with other psychiatric and neurological disorders, which is essential to establish the specificity of these findings for BPD. Another limitation is that the selection of only three measures (GAF, HAMA, NSSI) for correlational analysis with OCT and OCTA measurements may not fully capture the complex and multifaceted nature of BPD. While these measures provide valuable insights into general functioning, anxiety symptoms, and NSSI, they do not encompass all diagnostic criteria of BPD. This limitation restricts our ability to fully understand the potential relationships between retinal microvascular changes and the comprehensive spectrum of BPD symptoms. Future studies are encouraged to incorporate a broader range of diagnostic criteria and measures to provide a more holistic understanding of the association between OCT and OCTA findings and BPD. Additionally, the non‐specificity of GAF and HAMA and the focus on a single non‐diagnostic behavior by NSSI highlight the need for selecting measures that more specifically target the diagnostic features of BPD. Acknowledging these limitations, our findings should be interpreted with caution, and we advocate for further research to explore these associations with a more extensive set of BPD‐related measures. A significant limitation is the impact of various neurodegenerative conditions on the cognitive abilities of patients, which in turn may hinder their ability to cooperate during the acquisition of OCTA images. Consequently, this can result in images of suboptimal quality, potentially causing misinterpretations due to artifacts from movement Future research efforts will focus on designing studies that directly compare depression and BPD, alongside longitudinal and large‐scale studies to better understand these limitations.

## 5 CONCLUSION

While this study confirms that OCT and OCTA can non‐invasively detect microvascular and morphological changes in the retinas of BPD patients compared to healthy controls, it should be noted that these findings do not extend to a comprehensive diagnostic value for predicting or monitoring BPD symptoms. Further studies are needed to replicate these findings in BPD and compare them with other psychiatric disorders, including but not limited to major depressive disorder, bipolar disorder, and schizophrenia.

## FUNDING INFORMATION

This research was funded by the Youth Fund of the National Natural Science Foundation of China (82201702), the Youth Science Foundation of Xiangya Hospital: 2020Q20, and the Natural Science Foundation of Hunan Province (2020JJ4925).

## CONFLICT OF INTEREST STATEMENT

All authors declare no conflicts of interest.

## Supporting information


Table S1

## Data Availability

The datasets generated and/or analyzed during the current study are available from the corresponding author on reasonable request. Our patient is a patient with mental disorder. Based on ethics, privacy needs to be protected, so some information cannot be disclosed. Restrictions apply to the availability of some data, which were used under license for the current study, and so are not publicly available. Data are however available from the authors upon reasonable request and with permission.
