# Supplementary material for: The use of optical coherence tomography (OCT) and OCT angiography in borderline personality disorder compared to health control subjects
Source: CNS Neurosci Ther. 2024 Mar 26;30(3):e14699. doi: 10.1111/cns.14699 (PMC10964035; doi:10.1111/cns.14699)
Supplement: Supplementary file 1 — Table S1 [file CNS-30-e14699-s001.docx]

**Supplementary Table 1**. peripapillary capillary layer (RPCP) densities in the BPD and healthy controls (HC) eyes.

|  | BPD | HC | t | P-value |
| --- | --- | --- | --- | --- |
| Center | 6.3$\pm$3.88 | 6.8$\pm$3.13 | -0.750 | 0.455 |
| SI | 19.0$\pm$1.11 | 19.0$\pm$0.80 | -0.011 | 0.991 |
| II | $18.5\pm$1.47 | 18.7$\pm$1.31 | -0.595 | 0.553 |
| NI | 18.5$\pm$1.58 | 18.6$\pm$1.47 | -0.100 | 0.920 |
| TI | 17.7$\pm$3.67 | 17.9$\pm3.29$ | -0.433 | 0.666 |
| SO | 19.1$\pm$1.51 | 19.3$\pm$0.81 | -0.968 | 0.335 |
| IO | 19.3$\pm$1.59 | 19.3$\pm$0.96 | -0.233 | 0.816 |
| NO | 17.6$\pm$2.23 | $18.0\pm$1.61 | -1.197 | 0.234 |
| TO | 18.9$\pm$2.07 | 19.5$\pm$1.11 | -1.886 | 0.062 |
| Inner ring | 18.4$\pm$1.40 | 18.5$\pm$1.25 | -0.472 | 0.638 |
| Outer ring | 18.7$\pm$1.61 | 19.0$\pm$0.80 | -1.370 | 0.174 |

**Note:** independent samples t-test. SI, Superior inner; II, Inferior inner; NI, Nasal inner; TI, Temporal inner; SO, Superior outer; IO, Inferior outer; NO, Nasal outer; TO, Temporal outer.
